# Role and mechanism of CD90^+^ fibroblasts in inflammatory diseases and malignant tumors

**DOI:** 10.1186/s10020-023-00616-7

**Published:** 2023-02-06

**Authors:** Feng Zeng, Mengxiang Gao, Shan Liao, Zihua Zhou, Gengqiu Luo, Yanhong Zhou

**Affiliations:** 1 NHC Key Laboratory of Carcinogenesis, Hunan Cancer Hospital and The Affiliated Cancer Hospital of Xiangya School of Medicine, Central South University, Changsha, 410013 Hunan China; 2 Cancer Research Institute, Basic School of Medicine, Central South University, Changsha, 410078 Hunan China; 3 Department of Pathology, The Third Xiangya Hospital, Central South University, Changsha, 410013 Hunan China; 4 Department of Oncology, Loudi Central Hospital, Loudi, 417000 China; 5 Department of Pathology, Xiangya Hospital, Basic School of Medicine, Central South University, No. 88 of Xiangya Road, Changsha, 410008 Hunan China

**Keywords:** CD90, Fibroblasts, Inflammation, Fibrosis, Pathophysiology

## Abstract

Fibroblasts are highly heterogeneous mesenchymal stromal cells, and different fibroblast subpopulations play different roles. A subpopulation of fibroblasts expressing CD90, a 25–37 kDa glycosylphosphatidylinositol anchored protein, plays a dominant role in the fibrotic and pro-inflammatory state. In this review, we focused on CD90^+^ fibroblasts, and their roles and possible mechanisms in disease processes. First, the main biological functions of CD90^+^ fibroblasts in inducing angiogenesis and maintaining tissue homeostasis are described. Second, the role and possible mechanism of CD90^+^ fibroblasts in inducing pulmonary fibrosis, inflammatory arthritis, inflammatory skin diseases, and scar formation are introduced, and we discuss how CD90^+^ cancer-associated fibroblasts might serve as promising cancer biomarkers. Finally, we propose future research directions related to CD90^+^ fibroblasts. This review will provide a theoretical basis for the diagnosis and treatment CD90^+^ fibroblast-related disease.

## Introduction

Fibroblasts, as a type of mesenchymal cells constituting tissues and organs, secrete collagen and elastic fibers from the extracellular matrix (ECM), and play a key role in tissue fibrosis, autoimmunity, and wound healing (Koliaraki et al. 2020; Zhou et al. 2021). In inflammatory diseases, fibroblasts function as inflammatory cells, recruit leukocytes, drive angiogenesis, and promote chronic inflammation (Wei et al. 2021). In addition, fibroblasts affect the angiogenesis and immune evasion of tumor cells, play an important role in tumor invasion and metastasis, and affect drug resistance (Li et al. 2021).

With the development of single cell analysis techniques, it was found that fibroblasts are heterogeneous and the population of fibroblasts associated with disease varies. Fibroblasts can be classified into different subpopulations depending on their surface markers, such as thymocyte differentiation antigen-1(Thy-1)^+/−^ (CD90^+/−^) fibroblasts, FAPα^+/−^ fibroblasts (Croft et al. 2019), CD13^+/−^ fibroblasts, CD105^+/−^ fibroblasts (Kadefors et al. 2021), CD34^+/−^ fibroblasts (Mizoguchi et al. 2018), and α-SMA^+/−^ fibroblasts (Roelofs et al. 2017; Driskell et al. 2013). Among them, CD90^+^ fibroblasts are the key cell types involved in pathophysiological processes, such as the inflammatory response, fibrotic process, and cell proliferation and differentiation (Karpus et al. 2019). However, the biological functions of CD90^+^ fibroblasts vary in different tissues and organs (Schmidt et al. 2015). In terms of differentiation potential, only Thy-1^+^ (CD90^+^) myometrium and orbital fibroblasts are able to differentiate into myofibroblasts, only Thy-1^−^ (CD90^−^) myometrium and orbital fibroblasts are able to differentiate into adipose fibroblasts (Koumas et al. 2003), while in the lung, CD90^+^ fibroblasts inhibit their differentiation into myofibroblasts (Yang et al. 2020). In response to stimuli such as growth factors, CD90^−^ fibroblasts respond to stimulation with PDGF, IL-1β, IL-4, and increase transforming growth factor β (TGF-β) activity, Smad3 phosphorylation, α-smooth muscle actin, and fibronectin expression. Whereas CD90^+^ fibroblasts are resistant to stimulation by these factors (Zhou et al. 2004).CD90^+^ fibroblast activation depends on the involvement of the CD40 ligand pathway and TGF-β signaling (King et al. 2001), whereas the CD90^−^ subpopulation is not involved in the CD40 ligand pathway (Koumas et al. 2001). In wound healing, the differentiation of fibroblasts into myofibroblasts is an important component, and the fibroblast subpopulation that can differentiate into myofibroblasts is mainly CD90^+^ fibroblasts (CD90^−^ fibroblasts differentiate mainly into adipose fibroblasts), and the ECM of patients with diabetes mellitus (DM) exhibits fibroblast senescence, and their failure to differentiate to produce myofibroblasts leads to impaired wound healing (Kunkemoeller et al. 2017). In terms of the inflammatory response, CD90^+^ fibroblasts are highly amplified in rheumatoid arthritis (RA) and function to promote joint tissue inflammation (Mizoguchi et al. 2018; Varzideh et al. 2022; Kurose et al. 2022).

In summary, whether fibroblasts express CD90 or not affects their biological functions. Therefore, this review summarizes the relationship between CD90^+^ fibroblasts and cell proliferation and angiogenesis, as well as the role and possible mechanisms of CD90^+^ fibroblasts in diseases such as pulmonary fibrosis, inflammatory arthritis, and tumors, to provide a theoretical basis for a comprehensive understanding of the role and function of CD90^+^ fibroblasts.

## Main biological functions of CD90^+^ fibroblasts

### CD90^+^ fibroblast induce angiogenesis

**Fig. 1:**
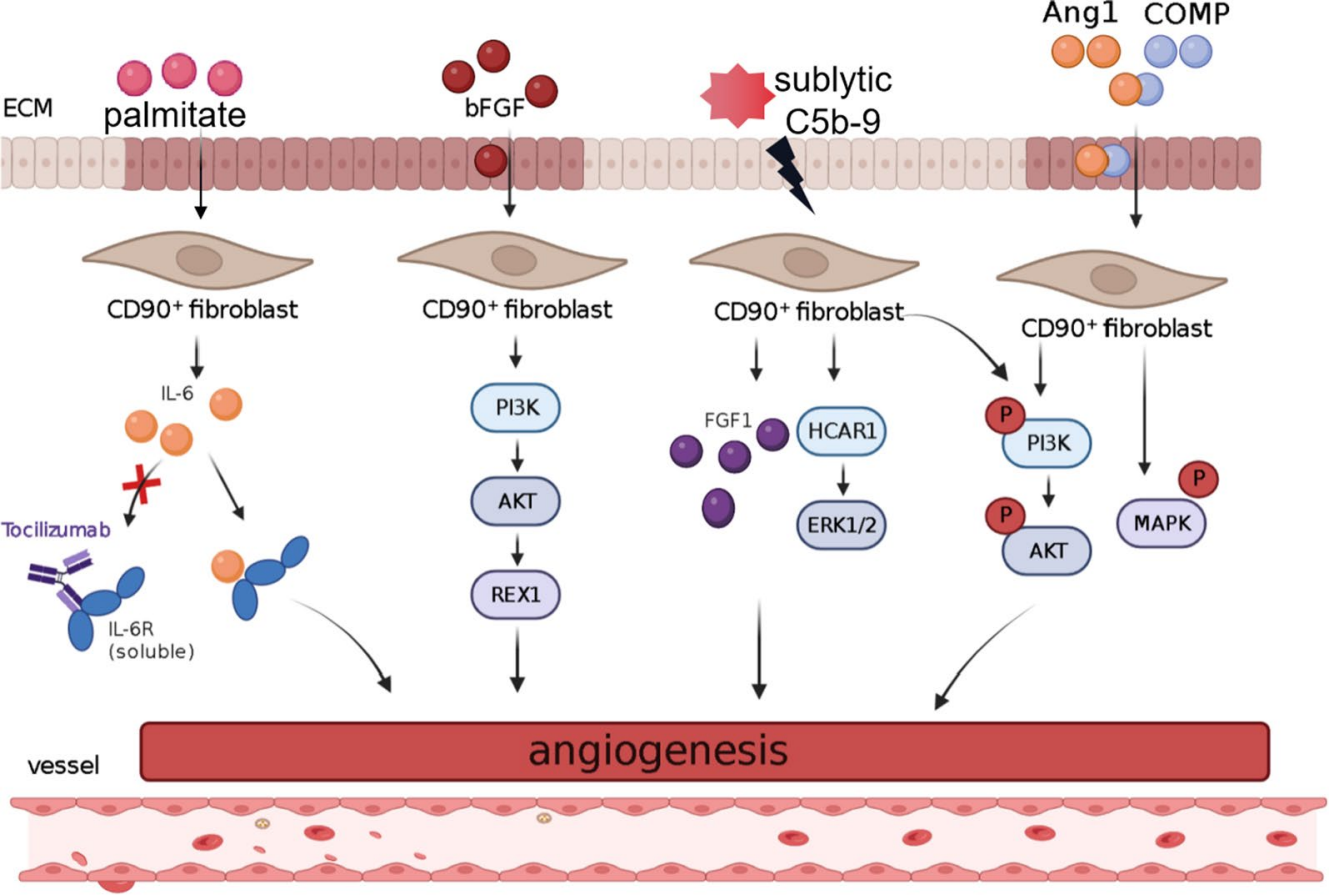
CD90^+^ fibroblasts induce angiogenesis by secreting growth factors and extracellular matrix precursors. CD90^+^ fibroblasts are the main source of IL-6, and IL-6 is involved in cell angiogenesis. In human deciduous deciduous teeth (SHEDs), bFGF induces angiogenesis through increased REX1 expression in the FGFR and Akt signaling pathways. At the same time, according to the existing research, bFGF can promote the proliferation of CD90^+^ fibroblasts. In the fibroblasts under the proliferation state, transcription factor REX1 is highly expressed. In addition, adipose-derived mesenchymal stem cells (AD-MSCs) can effectively secrete functional FGF1 and promote angiogenesis. In human fibroblast-like cells, cerebrovascular production is promoted by activating the lactate receptors HCAR1 and ERK1/2-PI3K/Akt signaling pathway. Meanwhile, in CD90^+^ periodontal ligament fibroblasts, angiopoietin-1 (Ang1) and cartilage oligomeric matrix protein (COMP) chimera COMP -Ang1 promote the cycle progression of human periodontal ligament cells and induce angiogenesis by mediating the phosphorylation of PI3K/Akt and MAPK

Fibroblasts maintain the structural integrity of connective tissue through the continuous secretion of growth factors and ECM precursors that are essential for the adhesion and spreading of endothelial cells (ECs), which are the main cells that form blood vessels. Therefore, fibroblasts might be involved in the induction of angiogenesis (Inoue et al. 2016; Chen et al. 2010; Zhou et al. 2022). Miyanaga et al. observed the thickening of perichondrium and the proliferation of vascular endothelial cells after one week of treatment with basic fibroblast growth factor (bFGF). While the number of CD44^+^ and CD90^+^ cartilage MSCs/progenitor cells increased, angiogenesis gradually increased. It reveals that the self-regeneration of CD90^+^MSCs may be the result of inducing angiogenesis (Miyanaga et al. 2018). While CD90^+^ fibroblasts are precisely differentiated from MSCs, their expression is closely related to the concentration of bFGF (Jia et al. 2018); therefore, CD90^+^ fibroblasts must play an imp1ortant role in angiogenesis, but the exact mechanism remains to be investigated. It has been reported that CD90^+^ fibroblasts are the main source of IL-6, which is a multi-effector cytokine involved in the tumor growth process (Huynh et al. 2016). Meanwhile, in CD90^+^ periodontal ligament fibroblasts, angiopoietin-1 (Ang1) and cartilage oligomeric matrix protein (COMP) chimera COMP-Ang1 promote the human periodontal ligament cell cycle process by mediating the phosphorylation of PI3K/Akt and MAPK (Lim et al. 2016). In summary, CD90^+^ fibroblasts are involved in tumor cell progression through the IL-6, PI3K/Akt and MAPK pathways. In addition, studies have shown that REX1 expression is enhanced by bFGF through the FGFR and Akt signaling pathways in human exfoliated deciduous teeth stem cells (SHEDs). IL-6 is involved in bFGF-induced REX1 expression in SHEDs, while the addition of IL-6 neutralizing antibody, FGFR, or Akt inhibitor pretreatment attenuated SHED-induced bFGF-induced REX1 expression (Nowwarote et al. 2017). REX1 is a surface marker of pluripotent stem cells (with the ability to differentiate into vascular endothelial progenitor cells). Besides, bFGF can promote the proliferation of CD90^+^ fibroblasts. and transcription factor REX1 is highly expressed in fibroblasts of proliferative state (Miyanaga et al. 2018; Du et al. 2017; Rieske et al. 2005; Singh et al. 2013). In human brain fibroblast-like cell, cerebrovascular generation is promoted by activate that lactic acid receptor HCAR1 and the ERK1/2-PI3K/Akt signaling pathway (Zhang et al. 2022). This suggested that during the angiogenesis induced by CD90^+^ fibroblasts, bFGF might participate in the regulation of angiogenesis mechanism through FGFR, IL-6 and Akt signaling pathways, which needs further investigation in the future. In another study, using adipose-derived mesenchymal stem cells (AD-MSCs) as a model, the microenvironment required for angiogenic proliferation was shaped by transfection with a fibroblast growth factor 1 (FGF1) plasmid, which was positive for CD90. In addition, FGF1-conditioned medium significantly increased the proliferation of NIH/3T3 cells (mouse embryonic fibroblasts) and human umbilical vein endothelial cell (HUVEC) tube formation. That is, AD-MSCs^FGF1^ (CD90 fibroblast dominant) effectively secreted functional FGF1 to promote angiogenic proliferation (Hoseini et al. 2017; Jacobs et al. 2016; Bourgine et al. 2019), suggesting that CD90^+^ fibroblasts in stromal cells might induce angiogenesis and promote cell proliferation by secreting multiple functional cytokines (Fig. 1).

**Fig. 2:**
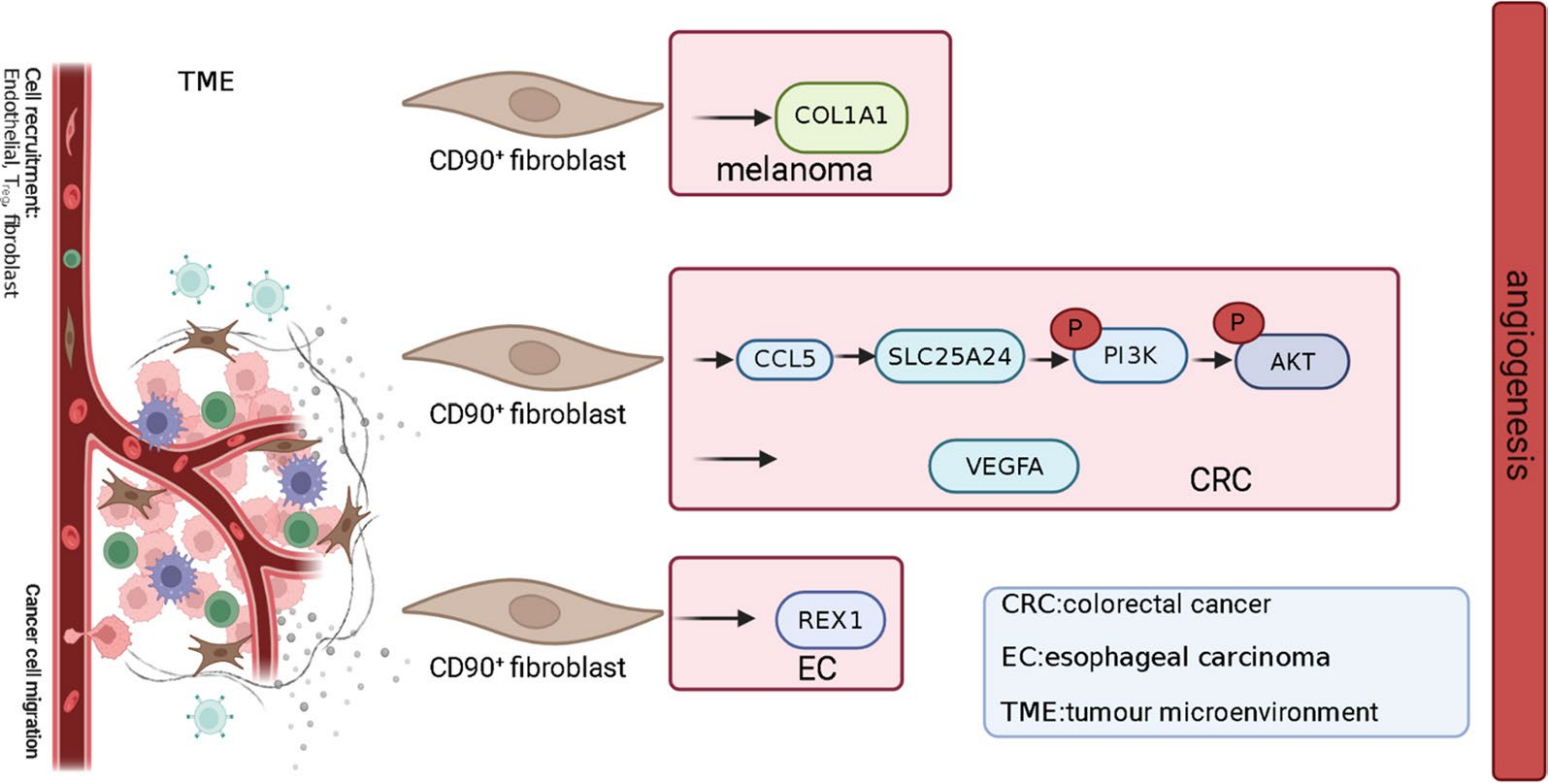
CD90^+^ fibroblasts induce angiogenesis in tumor microenvironment. Human CD90^+^ fibroblasts participate in angiogenesis of melanoma cells by secreting type I collagen. In colorectal cancer, chemokine ligand 5 (CCL5) positively regulates the expression of solute carrier family member (SLC25A24) in CD90^+^ fibroblasts, activates pAkt-pmTOR signaling, thus increasing the number of CD90^+^ fibroblasts, and promotes tumor angiogenesis by enhancing VEGFA expression and transdifferentiating fibroblasts into vascular endothelial cells. In esophageal carcinoma, CD90^+^ fibroblasts induce the up-regulation of angiogenesis-related genes

CD90^+^ fibroblasts play an important role in inducing angiogenesis in the tumor microenvironment, in addition to inducing angiogenesis in the normal physiological state. Goldstein et al. reported that normal human CD90^+^ fibroblasts enabled induce angiogenesis in melanoma cells through the secretion of type I collagen (Goldstein et al. 2005). CD90^+^ fibroblasts isolated from esophageal cancer tissues were found to have upregulated expression of angiogenesis-related genes (Krämer et al. 2020). In colorectal cancer, chemokine ligand 5 (CCL5) positively regulates the expression of a solute carrier family member (SLC25A24) in CD90^+^ fibroblasts and activates phosphorylated pAkt-pmTOR signaling, thereby increasing the number of CD90^+^ fibroblasts, and promoting tumor angiogenesis by enhancing VEGFA expression and transdifferentiating fibroblasts into vascular endothelial cells (Tancharoen et al. 2017; Gao et al. 2022). In conclusion, CD90^+^ fibroblasts induce angiogenesis in physiological processes and the tumor microenvironment by secreting relevant effector cytokines in combination with vascular endothelial cells, which has great application prospects for clinical regenerative medicine and tumor targeting therapy. Therefore, it is important to determine the possible mechanisms of CD90^+^ fibroblast-induced angiogenesis (Fig. 2).

### CD90^+^ fibroblasts affect tissue homeostasis

The balance between fibroblast proliferation, differentiation, and apoptosis is critical for the maintenance of tissue homeostasis, physiological wound healing, scar formation, prevention of tissue fibrosis, and tumor progression (Eckes et al. 2014). The expression of CD90 in fibroblasts balances the relevant organismal homeostases by influencing their own proliferation and differentiation. CD90 has been reported to maintain skin homeostasis by interacting with β3 integrins to inhibit dermal fibroblast proliferation and promote apoptosis. Mechanistically, the CD90-β3 integrin interaction induces upregulation of FasL (belonging to the tumor necrosis factor family) expression on dermal fibroblasts. This induces an extrinsic apoptotic pathway that activates the cystein cascade reaction and inhibits the proliferation of dermal fibroblasts, leading to an overall decrease in cell growth of CD90^+^ fibroblasts (Schmidt et al. 2016). In human lung CD90^+^ fibroblasts (LFs), the transcription factor STAT3 affects their phenotypic differentiation and function. STAT3 activation of LFs induced collagen I expression and had no significant effect on TGF-β, but inhibited α-SMA, CD90, and αvβ3 integrin expression. Inhibition of STAT3 signaling decreased CD90^+^ fibroblast resistance to astrosporine-induced apoptosis and the response to TGF-β in idiopathic pulmonary fibrosis. However, STAT3 inhibition increased α-SMA expression and restored β3 integrin expression in LFs dependent on ALK-5 and SMAD3/7-independent mechanisms (Pechkovsky et al. 2012). At the same time, PD-1 on CD4 + T cells promoted expression of STAT3 in human lung fibroblasts and mediated the production of IL-17A and TGF-β1 to promote pulmonary fibrosis (Celada et al. 2018). A lipophilic compound, cryptotanshinone, has also been found in Radix Salviae Miltiorrhizae root. It can inhibit STAT3 signaling pathway to prevent pulmonary fibrosis (Zhang et al. 2019). During renal fibrosis, discoid domain receptor 1 (DDR1) has been found to promote renal inflammation and fibrosis by promoting the phosphorylation of BCR and STAT3 (Borza et al. 2022). Inhibition of STAT3 in renal tubular epithelial cells prevents renal fibrosis (Zheng et al. 2019). The above results suggest that STAT3 transcription factor plays an important role in the CD90-mediated proliferation and differentiation of fibroblasts, as well as in the process of pulmonary fibrosis and renal fibrosis.

**Fig. 3:**
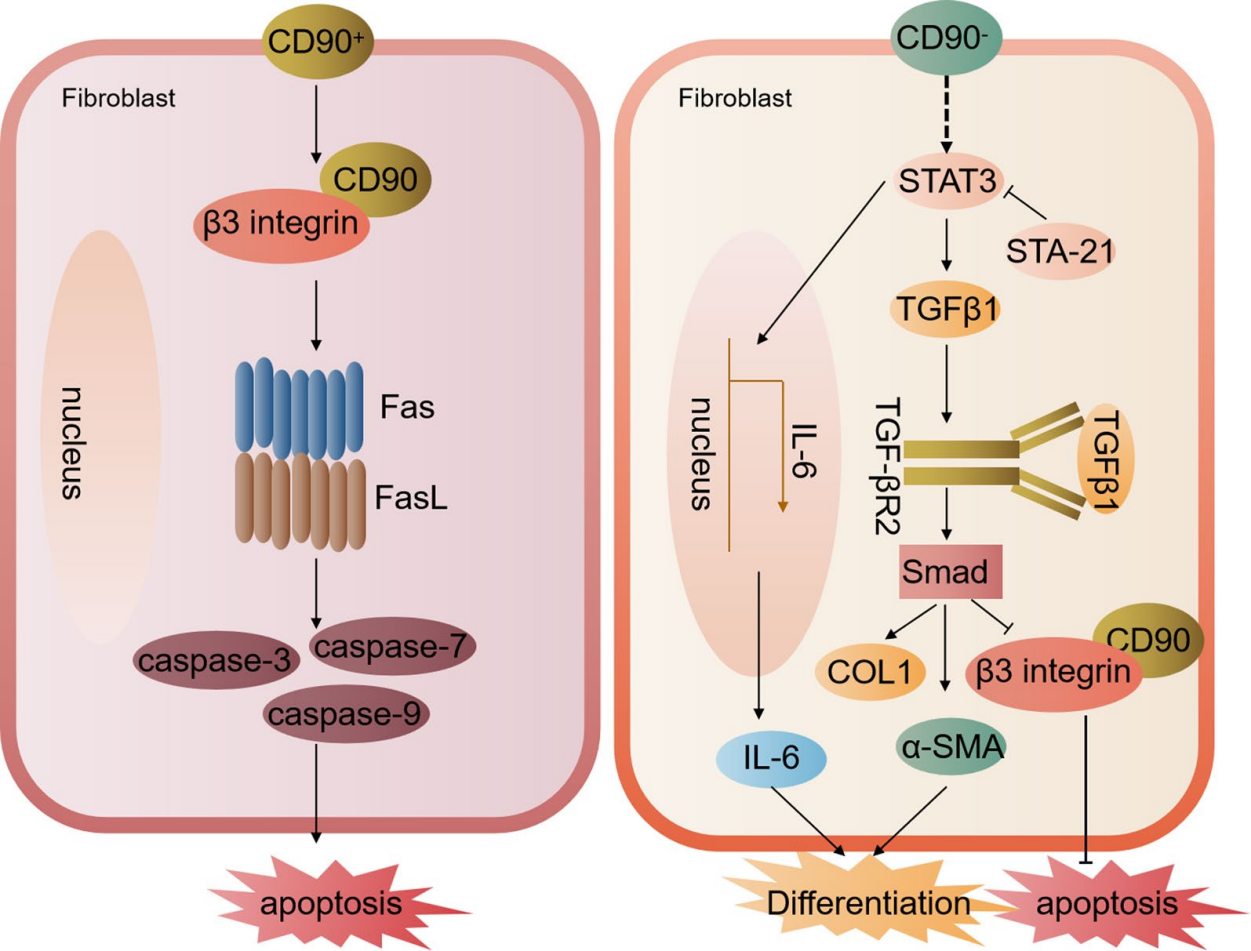
The mechanisms of CD90 expression in fibroblasts affecting its proliferation and differentiation. In human lung CD90^+^ fibroblasts (LFs), CD90 promotes fibroblast apoptosis and inhibits their differentiation into myofibroblasts by interacting with β3 integrins and upregulating the tumor necrosis family member Fas ligand FasL. This leads to the expression of apoptosis-related molecules caspase3/7/9. In human lung tissue with idiopathic pulmonary fibrosis (IPF), fibroblasts become negative for CD90 expression, in which STAT3 phosphorylation is activated, and STA-21 inhibits STAT3 activation, thereby activating TGFβ1-induced α-SMA and collagen I expression, thereby activating the differentiation potential of lung fibroblasts. However, at the same time, STAT3 activation inhibits CD90 and β3 integrin interaction, thereby inhibiting fibroblast apoptosis

The dynamic balance of the fibroblast differentiation process is also crucial for maintaining tissue homeostasis in vivo. CD90^+^ fibroblasts inhibit the differentiation of lung fibroblasts into myofibroblasts. In human normal fibroblasts, CD90 becomes positive, whereas myofibroblasts in fibroblastic lesions in human lung tissue from idiopathic pulmonary fibrosis (IPF) are CD90 negative. Exposure of human lung fibroblasts to inflammatory/fibrotic mediators (IL-1β, TNF-α, or FGF) induces a decrease of Thy-1 (CD90) expression (Yang et al. 2020), leading to TGF-β activation and TGF-β1 binding to its cell surface receptor, TGF-β receptor type II (TGF-βR2). This leads to phosphorylation of TGF-β receptor type I (TGF-β R1) and TGF-β1 recruitment, resulting in the formation of a heteropolymeric receptor complex. This complex phosphorylates Smad2 and Smad3, which bind to Smad4, leading to their nuclear translocation and the transcription of ACTA2 [encoding α smooth muscle actin (α-SMA)] (Yang et al. 2020; de la Mare et al. 2017). α-SMA then induces proliferation and differentiation of lung fibroblasts into CD90^−^ myofibroblasts. Myofibroblasts are considered to be the activated form of fibroblasts and can be distinguished from non-activated fibroblasts by the expression of α-SMA (Saada et al. 2006). Thus CD90^+^ lung fibroblasts have an inhibited ability to differentiate into myofibroblast cells, which has implications for tissue repair and remodeling processes (Fig. 3).

In addition, patients undergoing peritoneal dialysis (PD) for renal failure develop thickened peritoneal fibrosis, and researchers isolated the CD90^+^ subpopulation of human peritoneal fibroblasts (HPFB) and analyzed them according to a pro-fibroblastic myofibroblast signature. In healthy individuals, CD90^+^ cells constituted 45% of the HPFB population found in the greater omentum; however, the were not detected in the mural peritoneum. The number of CD90^+^ HPFBs was significantly increased in patients with PD, accounting for more than 70% and 95% of all HPFB found in the omentum and mural peritoneum, respectively. These data suggested that the fibrotic thickening of the peritoneum during PD might be caused by the expansion of CD90^+^ fibroblasts (Kawka et al. 2017). Thus, it is clear that CD90^+^ fibroblast-induced proliferation and differentiation play positive or negative roles in different tissues, sometimes promoting tissue repair and remodeling, but also promoting fibrotic thickening, leading to the development of disease.

## The role and mechanism of CD90^+^ fibroblasts in pulmonary fibrosis

The study of lung fibroblast heterogeneity is important because the lung is particularly susceptible to fibrosis caused by chemotherapy and radiation, inhaled particles, and systemic autoimmune diseases (Fries et al. 1994). In IPF, an aggressive and malignant pulmonary fibrotic disease, deletion of Thy-1 (CD90) expression in fibroblasts is associated with regions of active fibrogenesis, thus representing a pathologically relevant subpopulation of fibroblasts (Hardie et al. 2009). CD90 is a regulator of fibroblast stiffness sensing and is physically coupled to inactive αvβ3 integrins through its RGD-like motif. CD90 alters the affinity of baseline integrins for ECM ligands and promotes pre-adhesive aggregation of integrins and membrane rafts via the glycophosphatidylinositol tether of CD90. Disruption of CD90-αvβ3 coupling alters the recruitment of Src family kinases to the adhesion complex and impairs mechanosensitivity-induced Rho signaling and stiffness sensing (Fiore et al. 2015), leading to fibrotic disease. CD90 differential expression affects fibroblast proliferation and fibrotic signaling. In IPF, myofibroblasts proliferating within fibroblastic lesions are CD90^−^, whereas normal lung fibroblasts are predominantly CD90^+^, and CD90^+^ and CD90^−^ subsets have different functional properties (Sanders et al. 2007). In rat lung fibroblasts, the CD90^−^ subset activates TGF-β and expresses α-SMA in response to fibrotic stimuli, whereas the CD90^+^ subset does exert this function (Zhou et al. 2004).

Thy-1/CD90 has an effect on lung fibroblast proliferation and fibrotic signaling. In acute interstitial pneumonia (AIP), Thy-1 (CD90) expression; the expression of MMP-2, Occludin, α-SMA, wave proteins and β-linked proteins; and the phosphorylation of β-linked protein were significantly reduced. This indicated WNT pathway inactivation and reduced lung fibrosis, while cell proliferation was inhibited and apoptosis was accelerated. These results suggested a potential role for Thy-1 (CD90) inactivation of the WNT pathway in AIP remission. Inactivation of the WNT signaling pathway could alleviate pulmonary fibrosis by reducing lung fibroblast proliferation in AIP (Chen et al. 2019). This suggested that Thy-1^+^ (CD90^+^) fibroblasts can inhibit pulmonary fibrosis and delay the onset of lung disease through the WNT signaling pathway. Furthermore, stimulation of lung fibroblasts with lipopolysaccharide (LPS) suppressed Thy-1 (CD90) expression and upregulated integrin β3 (Itgb3) to activate the PI3K-Akt-mTOR pathway and inhibit lung fibroblast autophagy. When Thy-1 (CD90) was overexpressed or integrin β3 was inhibited, LPS-induced autophagy inhibition and lung fibrosis were prevented (Wan et al. 2019). This suggested that CD90^+^ fibroblasts might play an inhibitory role in pulmonary fibrosis; however, the exact mechanism is mostly unknown. It has been reported that Thy-1 (CD90) deficiency enhances cellular contractility-driven strain sclerosis in the temporary ECM in vitro and leads to elevated αvβ3 integrin activation, increased fibrosis, and increased mortality after fibrotic lung injury in vivo. These data suggested that αvβ3 integrin and temporary ECM drive progressive fibrosis through physical sclerosis of the fibrotic ecotone (Fiore et al. 2018).

**Fig. 4:**
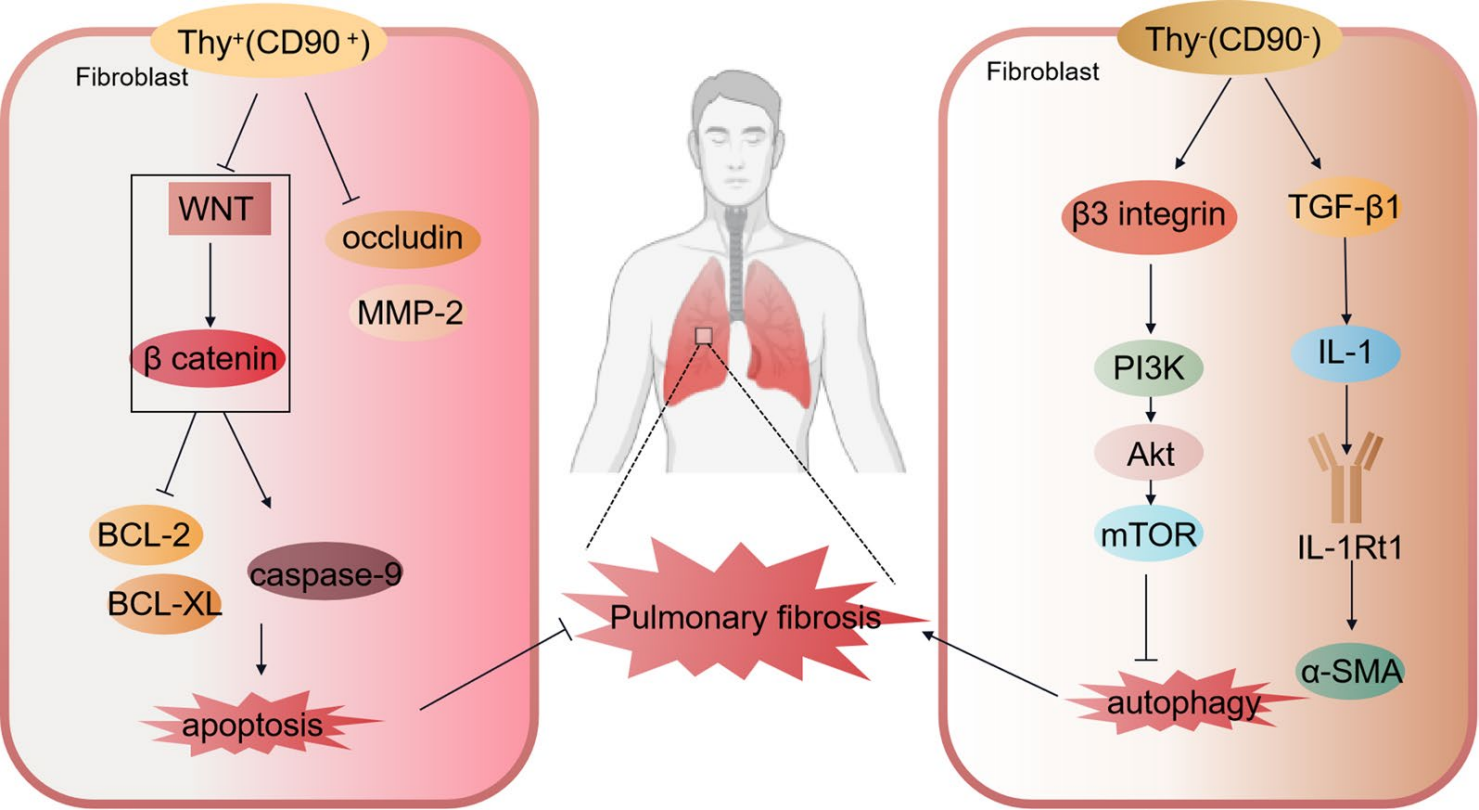
The role and mechanism of CD90 fibroblasts in pulmonary fibrosis. Thy-1 overexpression, significant decrease in the expression of MMP-2, Occludin, α-SMA, wave proteins, and β-linked protein; and phosphorylation of β-linked protein, i.e. inactivation of the WNT pathway, thereby decreasing lung fibrosis. Thy-1 expression was associated with downregulation of the anti-apoptotic molecules Bcl-2 and Bcl-xL, and an increase in caspase-9 levels. Thy-1-pulmonary fibroblasts are resistant to apoptosis. Stimulation of lung fibroblasts with lipopolysaccharide (LPS) inhibits Thy-1 expression and upregulates integrin β3 (Itgb3) to activate the PI3K-Akt-mTOR pathway and inhibit lung fibroblast autophagy. When Thy-1 is overexpressed or integrin β3 is inhibited, LPS-induced autophagy inhibition and lung fibrosis are prevented. Thy-1-pulmonary fibroblasts secrete twice as much TGF-β as the Thy-1^+^ subset, and TGF-β1 significantly upregulates IL-1Rt1 expression in Thy-1-fibroblasts. TGF-β and interleukin-1 (IL-1) are important players in the development of pulmonary fibrosis

These observations indicated that CD90^+^ and CD90^−^ fibroblast subpopulations differ in the regulation of lung fibrosis. Thy-1^+^ (CD90^+^) fibroblast cell lines synthesize two to three times more collagen than Thy-1^−^ (CD90^−^) lines (Derdak et al. 1992). Thy-1 (CD90) expression was associated with downregulation of the anti-apoptotic molecules Bcl-2 and Bcl-xL and an increase in caspase-9 levels, and Thy-1^−^ (CD90^−^) lung fibroblasts are resistant to apoptosis (Liu et al. 2017; Hagood et al. 2005). Thy-1^+^ (CD90^+^) and Thy-1^−^ (CD90^−^) lung fibroblast subsets also differ in the level of transforming growth factor-β (TGF-β) isoforms expressed, with Thy-1^−^ (CD90^−^) lung fibroblasts secreting twice as much TGF-β as the Thy-1^+^ (CD90^+^) subset. TGF-β1 significantly downregulates IL-1Rt1 expression in Thy-1^+^ (CD90^+^) fibroblasts, but not in Thy-1^−^ (CD90^−^) fibroblasts. IL-1RtI downregulation in Thy-1^+^ (CD90^+^) fibroblasts decreased the response to IL-1-mediated induction of IL-6 protein synthesis. In contrast, TGF-β and interleukin-1 (IL-1) are important players in the development of lung fibrosis, further suggesting an inhibitory role of Thy-1^+^ (CD90^+^) fibroblasts in the process of lung fibrosis (Silvera et al. 1994; Tan et al. 2019). In summary, there is heterogeneity in CD90^+^ and CD90^−^ fibroblast subpopulations, and CD90^+^ fibroblasts rely on αvβ3 integrin and WNT signaling pathways to alleviate pulmonary fibrosis and inhibit the development of lung inflammation. In contrast, CD90^−^ fibroblasts activate TGF-β and express α-SMA in response to fibrotic stimuli, inducing myofibroblast production and inhibiting fibroblast autophagy and apoptosis, leading to the development of pulmonary fibrosis (Fig. 4).

## The role and mechanism of CD90^+^ fibroblasts in inflammatory arthritis

The synovium is a mesenchymal tissue composed mainly of fibroblasts, and in RA, synovial tissue displays a significant increase in proliferation, inflammation, and invasiveness, and destroys joints, in which a subset of CD90 fibroblasts located in the inferior layer expand significantly. However, the molecular mechanisms of differentiation and expansion of these fibroblasts in RA remain unknown. In the synovial fibroblast population, the CD90^+^ HLA-DR^+^ fibroblast subset expresses most IL-6, and high levels of CXCL12 and interferon-stimulating factor (ISG), indicating that RA characterized by CD90^+^ fibroblasts is highly inflammatory (Zhang et al. 2019). It has also been reported that FAP^+^ CD90^+^ fibroblasts, associated with inflammation, undergo significant amplification, and that successive transfer of synovial CD90^+^ synovial lining fibroblasts exacerbates joint inflammation(Croft, Campos, Jansen, Turner, Marshall, Attar, Savary, Wehmeyer, Naylor, Kemble, Begum, Dürholz, Perlman, Barone, McGettrick, Fearon, Wei, Raychaudhuri, Korsunsky, Brenner, Coles, Sansom, Filer and Buckley 2019). Wei et al. found that in active RA, the expression levels of NOTCH3 and NOTCH target genes were significantly upregulated in synovial CD90^+^ fibroblasts, and that genetic deletion of *NOTCH3* or monoclonal antibody blockade of NOTCH3 signaling attenuated inflammation and prevented joint damage in inflammatory arthritis. This suggested that CD90^+^ fibroblasts are regulated by endothelial-derived NOTCH signaling and that this matrix crosstalk pathway underlies the inflammation and pathology of inflammatory arthritis (Wei et al. 2020; Onuora 2020). Fibroblast-like synoviocytes (FLS) in RA express high levels of CD90, IL-1RI, and IL-1β (Huang et al. 2009). In arthritic synovial tissues, pro-inflammatory cytokines, such as IL-1β and tumor necrosis factor alpha (TNF-α), enhanced bone morphogenetic protein 2 (BMP-2) and 6 ((BMP-6) expression in FLS in vitro. Moreover, BMP-2 promoted FLS (CD90^+^ fibroblasts) cell apoptosis, while BMP-6 prevented nitric oxide-induced FLS cell apoptosis. The results suggested that BMP-2 and BMP-6 are expressed in the arthritic synovium, are regulated by pro-inflammatory cytokines, and differentially regulate CD90 fibroblast-like synovial cell apoptosis (Lories et al. 2003). Thus BMP-2 and BMP-6 are important signaling molecules for the regulation of inflammatory arthritis by CD90 fibroblasts.

Different fibroblast subpopulations drive inflammation and injury in arthritis differently. Single-cell transcriptional analysis identified two distinct fibroblast subpopulations in the fibroblast activating protein-α (FAPα)^+^ population. Immune effector fibroblasts of FAPα^+^ Thy-1^+^ (CD90^+^) located in the synovial sublining, and destructive fibroblasts of FAPα^+^ Thy-1^−^ (CD90^−^) restricted to the synovial lining. When relayed into the joint, FAPα^+^ Thy-1^−^ (CD90^−^) fibroblasts selectively mediated bone and cartilage damage, with little effect on inflammation, whereas transfer of FAPα^+^ Thy-1^+^ (CD90^+^) fibroblasts resulted in more severe and persistent inflammatory arthritis, with minimal effect on bone and cartilage. These results suggested that anatomically discrete and functionally distinct subpopulations of fibroblasts with non-overlapping functions are important for cell-based therapies aimed at modulating inflammation and tissue damage (Croft et al. 2019). The important role of CD90^+^ fibroblasts in immune inflammatory arthritis is also further illustrated.

## CD90^+^ fibroblasts are involved in inflammatory skin diseases and skin scar formation

Scarring is a fibrotic response to wounds designed to restore barrier integrity, in which a subpopulation of CD90-expressing fibroblasts plays an important role (Hagood et al. 2005). The abundant CD34^+^ dermal fibroblasts present in healthy human skin are lost in the skin of patients with systemic sclerosis, and CD34^−^, podoplanin^+^, and CD90^+^ fibroblasts are present. This transition is limited to the upper dermis in several inflammatory skin diseases; however, in systemic sclerosis, it can occur in all areas of the dermis (Nazari et al. 2016). CD90^+^ fibroblasts have been shown to be generated and replaced by a steady-state CD34^+^ network in scleroderma, and the findings suggested that most scars exhibit a CD90diffuse/CD34negative/minority pattern, and dual CD90^+^ /CD34^+^ fibroblasts were observed in 91% of scars. In reparative scars, CD90 expression was reversed to a CD34^+^ /CD90^−^ mature state. Pathological scars exhibit prolonged CD90 expression. Both CD90^+^ and SMA^+^ fibroblasts collagenize the scars, with CD90^+^ fibroblasts being more prevalent. This suggests that CD90^+^ fibroblasts might be an important player in skin scar formation (Ho et al. 2019; Zhao et al. 2016; Hardy 1989). Junnan et al. isolated hypertrophic scar-derived fibroblasts (HSFs; CD90^+^ fibroblasts) and celiac fat-derived stem cells (CFSCs) from individual patients and found that CFSCs differentiated into adipocytes and osteoblasts under appropriate induction conditions, and the conditioned medium (CM) of CFSCs inhibited HSF (CD90^+^ fibroblasts) proliferation and migration. The results suggest that CFSCs are associated with inhibition of fibrosis in HSF (CD90^+^ fibroblasts) through paracrine effects, and the use of CFSC-CM might be a new therapeutic strategy for hypertrophic scars (Chen et al. 2019). This also suggested that CD90^+^ fibroblasts are associated with the fibrotic state during skin scar formation.

Perivascular accumulation of lymphocytes might be a prominent histopathological feature of various inflammatory skin diseases, such as systemic sclerosis, cavernous dermatitis, and cutaneous lupus, which are often associated with lymphocyte perivascular outer membrane fibroblast (CD90^+^ fibroblast) status (Barron et al. 2019). Fibrotic dermatoses are characterized by excessive proliferation of fibroblasts and excessive accumulation of ECM. Among these, there is heterogeneity of fibroblasts in keloids (a paradigm of fibrotic dermatosis), with a significantly increased percentage of mesenchymal fibroblast subpopulations (CD90^+^ fibroblasts) in keloids compared with normal tissue. Functional studies suggest that mesenchymal fibroblasts (CD90^+^ fibroblasts) are essential for collagen overexpression in keloids. An increased subpopulation of mesenchymal fibroblasts was also found in another fibrotic skin disease, scleroderma, suggesting that this is a widespread mechanism of inflammatory skin fibrosis (Deng et al. 2021; Marangoni et al. 2022). In response to injury, tissue stretch, and cytokines (e.g., TGF-β), CD90^+^ fibroblasts are activated and then differentiate into myofibroblasts. Once induced, myofibroblasts produce and secrete higher levels of ECM proteins, including multiple types of collagen, and express contractile proteins, such as αSMA, which underlie their ability to contract and close injured areas (Frangogiannis 2017).

## CD90^+^ fibroblasts influence the disease process of malignant tumors

### CD90^+^ fibroblasts promote pancreatic cancer invasion and metastasis

Cancer stem cells and the tumor microenvironment (TME) are responsible for chemotherapy resistance, cancer cell proliferation, and metastasis. CD90 expression has been identified in cancer stem cells, as well as in the highly aggressive cancer microenvironment (Kumar et al. 2016). In pancreatic cancer, 60–70% of the tumor mass consists of stromal tissue characterized by cancer associated fibroblasts (CAFs) and the excessive deposition of collagen and other ECM components (Ziani et al. 2018; Öhlund et al. 2014). CD90^+^ CAFs are the predominant stromal cell type and are one of the most critical components of the TME. They are characterized by a highly heterogeneous cellular origin (originating from fibroblasts and endothelial cells) and α-SMA expression, playing an important role in tumor invasion and metastasis (Goicoechea et al. 2014). In pancreatic cancer (PC), MRC-5 (human embryonic lung fibroblasts) inhibits PC cell colony-forming capacity, cell migration, and invasive potential. MRC-5 also induces S-phase cell cycle arrest, but does not enhance PC cell apoptosis. This was associated with reduced CD90 expression in culture conditions (Ding et al. 2018). CD90 is significantly overexpressed in pancreatic adenocarcinoma (PDAC) and its metastatic cancers, while it was negative in the normal pancreas and 82.7% of adjacent normal pancreatic tissues. Moreover, CD90 expression was mainly present in the PDAC stroma, comprising fibroblasts and vascular endothelial cells, which could be a promising marker to distinguish pancreatic adenocarcinoma from the normal pancreas and non-malignant pancreatic disease. Double immunostaining of CD90 with CD24 [a cancer stem cell (CSC) marker of PDAC] revealed that CD90^+^ fibroblasts were clustered around CD24^+^ malignant ducts, indicating that CD90^+^ fibroblasts are involved in tumor-stromal interactions, promote the development of PDAC, and could be a promising marker for this cancer (Zhu et al. 2014). Based on this property, Bam et al. developed a Thy-1-targeted microbubble (MBThy1) ultrasound contrast agent that specifically identified overexpression of Thy-1 in the vascular system of mouse PDAC tissues using ultrasound (US) imaging. They also constructed a clinically translatable MB Thy-1-scFv using a single-stranded variable fragment (scFv) site-specific biocoupling approach. As assessed by confocal microscopy, the scFv showed highly specific binding to VEGFR2-positive vascular systems and fibroblast-like matrix components around human PDAC tissue conduits (Bam et al. 2020). This is promising for planning further clinical development of the Thy-1-targeted contrast agent MB for the early and accurate diagnosis of human PDAC using molecular imaging, thus improving the overall survival of patients with this fatal cancer. In conclusion, CD90^+^ fibroblasts play an important role in the malignant biological behavior of PC and its development. They are also promising biomarkers for PC. These discoveries have provided a theoretical basis for the development and application of clinical diagnostic and therapeutic tools for pancreatic cancer, which has far-reaching implications.

### CD90^+^ fibroblasts are involved in the development of prostate cancer

There is growing evidence that cancer-associated stromal cells play an important role in both tumor progression and carcinogenesis. Prostate cancer is surrounded by a layer of stromal CD90^+^ fibroblasts (Sauzay et al. 2019; Kwon et al. 2019). Supernatants of prostate tissue cultures digested by collagenase, which contain proteins made by cells within the tissue, were collected. Subsequent quantitative proteomic analysis showed that increased N-glycosylated protein CD90/Thy-1 was detected in the cancer supernatant and CD90^+^ stromal fibroblasts were identified in the tumor gland using immunohistochemistry (True et al. 2010). CD90^+^ fibroblasts are involved in ECM remodeling and in the genetic differential induction of the cysteine protein (RECK) pathway in the pluripotent embryonal carcinoma cell line NCCIT. Mechanistically, MMP synthesized by CD90^+^ fibroblast stromal cells leads to ECM degradation, which in turn promotes tumor cell escape, a process facilitated by reduced TIMP expression and downregulation of RECK in the prostate cancer process (Pascal et al. 2011). These results suggest that Thy-1 (CD90) is a potential marker for prostate cancer and could be one of the therapeutic targets for prostate cancer in the clinical setting. CD90^+^ fibroblasts are involved in the oncological process of prostate cancer, which provides a theoretical basis for the diagnosis and treatment of prostate cancer and represent a promising biomarker for prostate cancer.

### CD90^+^ fibroblasts are involved in the oncological progression of hepatocellular carcinoma

CD90 is mainly expressed in CAFs. Zhao et al. found that CD90 expression was significantly higher in hepatocellular carcinoma (HCC) tissues than in adjacent non-tumor and normal liver tissues. CD90 was all derived from CAFs in HCC tissues, and CD90 expression in CAFs was associated with pathological grade, satellite lesions, and portal vein carcinoma thrombosis. The combination of CD90 and OCT4, which is highly expressed in HCC, is more sensitive and improves the predictive accuracy as a prognostic factor for HCC (Zhao et al. 2016). In JHH6 HCC cells, CD90^+^ cells showed a high proliferation rate. However, markers such as CD44, CD29, CD105, CD166, CD54, CD106, and OCT4 were not differentially expressed between CD90^+^ and CD90^−^ populations. Other cancer-related genes, such as *HGF* (encoding hepatocyte growth factor), *FASP* (encoding fibroblast-associated protein), and *ACTA2* (encoding alpha smooth muscle actin 2) were highly expressed in CD90^+^ cells (Sukowati et al. 2013). This suggested that CD90^+^ cancer-associated fibroblasts promote the proliferation of HCC cells, are involved in the oncological progression of HCC, and could be a candidate marker for tis cancer (Ding et al. 2019). CD90^+^ cancer-associated fibroblasts are highly involved in the development of HCC through the secretion of cytokines and angiogenic factors, such as CAFs, which express high levels of CD90 and are enriched in HCC tissues. Placental growth factor (PlGF), which is significantly associated with CD90 expression, was significantly correlated, and high levels of both PlGF and CD90 were associated with tumor angiogenic markers (CD31, CD34, and CD105 (Liu et al. 2020). This suggests that CD90^+^ cancer-associated fibroblasts can produce PlGF and might provide an effective target for CD90^+^ cancer-associated fibroblast-regulated HCC neoangiogenesis.

## Conclusion and future directions

Although CD90 has been widely studied as a cancer cell surface signaling molecule and a cancer stem cell surface marker, its stemness characteristics and cancer-promoting mechanisms are less well studied. However, CD90 (Thy-1) is a representative molecule in fibroblasts, and the CD90 fibroblast subpopulation is involved in a variety of human pathophysiological processes (Table 1), and has provided theoretical evidence for the development of diagnostic and therapeutic tools for clinical diseases. Therefore, it is important to study the role of the CD90^+^ fibroblast subpopulation in human disease processes and their possible mechanisms. CD90^+^ fibroblasts are involved in regulating various physiological processes, such as the induction of angiogenesis and influencing tissue homeostasis. CD90^+^ fibroblasts also stimulate in situ angiogenesis by regulating capillary morphogenesis in the stromal cell microenvironment. Mechanistically, it may be that in CD90^+^ fibroblasts, bFGF binds to the fibroblast growth factor receptor (FGFR), thereby activating the MAPK and Akt signaling pathways, stimulating IL-6 transcription, and leading to upregulation of REX1 to induce angiogenesis; however, this needs to be confirmed by further studies. CD90 expression in fibroblasts is involved in skin repair and tissue remodeling processes by affecting their own proliferation and differentiation, thus maintaining tissue homeostasis. In CD90^+^ fibroblasts, CD90 promotes apoptosis by interacting with β3 integrin to promote the expression of the apoptosis-related molecule caspase-9, thereby promoting fibroblast apoptosis. In addition, some CD90^+^ fibroblasts have the potential to differentiate into osteoblasts and adipocytes in vitro. These observations have implications for skin repair and tissue remodeling. However, in HPFB, the expansion of CD90^+^ fibroblasts might lead to fibrotic thickening of the peritoneum during overlay dialysis in patients. Thus, CD90^+^ fibroblast-induced proliferation and differentiation play positive and negative roles in different tissues and might promote either tissue repair and remodeling or fibrotic thickening, leading to disease.

**Table 1:** Physiological functions and cytokines related to the induction of CD90^+^/CD90^−^ fibroblasts in some cells and disease

Cell name	CD90^+^/CD90^−^	Cytokines	Functions	References
Myometrial and orbital fibroblasts	CD90^+^	–	Differentiate into myofibroblasts	Koumas et al. (2003)
	CD90^−^		Differentiate into adipose fibroblasts	
Lung fibroblast	CD90^+^	PDGF, IL-1β, IL-4, TGF-β, Smad3, α-SMA	Inhibit differentiation into myofibroblasts	Yang et al. (2020)
	CD90^−^		Differentiate into myofibroblasts	
Embryo fibroblast	CD90^+^	FGF1	Inducing angiogenesis	Hoseini et al. (2017), Jacobs et al. (2016), Bourgine et al. (2019)
Melanoma fibroblasts	CD90^+^	COL1A1	Inducing angiogenesis	Goldstein et al. (2005)
Esophageal cancer fibroblasts	CD90^+^	FAP, SMA, COL1A1, COL3A1	Inducing angiogenesis	Krämer et al. (2020)
Colorectal cancer fibroblasts	CD90^+^	CCL5, SLC25A24, pAkt, pmTOR	Inducing angiogenesis	Tancharoen et al. (2017), Gao et al. (2022)
Dermal fibroblasts	CD90^+^	ITGβ3, FasL	Promote apoptosis and maintain skin homeostasis	Schmidt et al. (2016)
Peritoneal fibroblasts	CD90^+^	TGF-β1	Promote cell proliferation, leading to fibrotic thickening of the peritoneum	Kawka et al. (2017)
Synovioblast	CD90^+^	IL-6, CXCL12, ISG, NOTCH3, IL-1RI, IL-1β	Inducing high inflammation	Huang et al. (2009)

CD90^+^ fibroblasts are involved in various pathological processes, such as the induction of pulmonary fibrosis, regulation of inflammatory arthritis, involvement in inflammatory skin diseases, and skin scar formation, and can be used as biomarkers for related cancers (Table 2). CD90^+^ fibroblasts are most widely studied in inflammatory arthritis, where synovial tissue is the main site of action, and CD90^+^ fibroblasts are mainly located in the subsynovial layer, i.e. the lining layer. In RA, expansion of CD90^+^ fibroblasts exacerbates inflammation. Among pulmonary fibroblasts, CD90^+^ fibroblasts alleviate pulmonary fibrosis by inhibiting the WNT signaling pathway. However, the main mechanisms of action of CD90^+^ fibroblasts in both arthritis and pulmonary fibrosis remain to be fully described.

**Table 2:** Relevant fibroblasts (CD90^+^/CD90^−^) involved in some diseases

Fibroblasts (CD90^+^/CD90^−^)	Disease name	References
CD90^+^	Rheumatoid arthritis	Mizoguchiet al. (2018), Varzideh et al. (2022), Kurose et al. (2022)
	Inflammatory arthritis	Wei et al. (2020), Onuora (2020)
	Inflammatory skin disease	Nazari et al. (2016), Barron et al. (2019)
	Pulmonary fibrosis	Pechkovsky et al. (2012), Zhang et al. (2019)
	Melanoma	Goldstein et al. (2005)
	Esophageal cancer	Krämer et al. (2020)
	Colorectal cancer	Tancharoen et al. (2017), Gao et al. (2022)
	Renal fibrosis	(Borza et al. 2022; Zheng et al. 2019)
	Prostatic cancer	Sauzay et al. (2019), Kwon et al. (2019)
	Pancreatic cancer	Ziani et al. (2018), Öhlund et al. (2014), Ding et al. (2018)
	Liver cancer	Zhao et al. (2016)
CD90^−^	Pulmonary fibrosis	Celada et al. (2018), Zhang et al. (2019), Yang et al. (2020)

In conclusion, CD90^+^ fibroblasts are a double-edged sword involved in human disease processes and can be applied in clinical diagnosis and therapy, and regenerative medicine. Current research on CD90^+^ fibroblasts focuses on their involvement in inflammatory responses, their stemness characteristics, and their potential as cancer biomarkers. However, the specific molecular mechanisms have not been fully described; therefore, future research should focus on the specific molecular mechanisms of CD90^+^ fibroblasts in different tissues, organs, and cancer cells to provide a theoretical basis for the development of precise therapeutic protocols and targeted therapies in the clinical setting.

## Data Availability

Not applicable.
